# Krüppel-like factor 12 is a novel negative regulator of forkhead box O1 expression: a potential role in impaired decidualization

**DOI:** 10.1186/s12958-015-0079-z

**Published:** 2015-07-30

**Authors:** Hui Zhang, Xudong Zhu, Jing Chen, Yue Jiang, Qun Zhang, Chengcai Kong, Jun Xing, Lijun Ding, Zhenyu Diao, Xin Zhen, Haixiang Sun, Guijun Yan

**Affiliations:** Reproductive Medicine Center, Drum Tower Clinic Medical College of Nanjing Medical University, Nanjing, 210029 Jiangsu China; College of Science Isotope Laboratory, Nanjing Agricultural University, Nanjing, 210095 Jiangsu China; Department of Obstetrics and Gynecology, Reproductive Medicine Center, Nanjing Drum Tower Hospital, Nanjing University Medical School, Nanjing, 210008 Jiangsu China

**Keywords:** KLF12, FOXO1, Decidualization, RIF

## Abstract

**Background:**

Decidualization is a prerequisite for successful implantation and the establishment of pregnancy. Krüppel-like factor 12 (KLF12) is a negative regulator of endometrial decidualization *in vitro*. We investigated whether KLF12 was associated with impaired decidualization under conditions of repeated implantation failure (RIF).

**Methods:**

Uterine tissues were collected from a mouse model of early pregnancy and artificial decidualization for immunohistochemistry, Western blot and real-time PCR analysis. Reporter gene assays, chromatin immunoprecipitation-PCR and avidin-biotin conjugate DNA precipitation assays were performed to analyze the transcriptional regulation of forkhead box O1 (FOXO1) by KLF12. Furthermore, the protein levels of KLF12 and FOXO1 in patients with RIF were analyzed by Western blot and immunohistochemistry.

**Results:**

KLF12 led to defective implantation and decidualization in the mouse uterine model of early pregnancy and artificial decidualization by directly binding to the FOXO1 promoter region and inhibiting its expression in human endometrial stromal cells. Elevated KLF12 expression was accompanied by decreased FOXO1 expression in the endometria of patients with RIF.

**Conclusions:**

As a novel regulator, KLF12 predominantly controls uterine endometrial differentiation during early pregnancy and leads to implantation failure.

**Electronic supplementary material:**

The online version of this article (doi:10.1186/s12958-015-0079-z) contains supplementary material, which is available to authorized users.

## Background

Stromal decidualization in the endometrium is crucial for successful embryo implantation and the maintenance of pregnancy. This process is defined by the mesenchymal-to-epithelial transformation of endometrial fibroblasts and stromal cells into secretory epithelioid decidual cells, which affect endometrial receptivity [1]. In mice and rats, mechanical or chemical stimulation of the endometrium is required to induce decidual transformation. In contrast with what occurs in many species, decidualization of the human endometrial stroma begins spontaneously during the late luteal phase of the menstrual cycle, even in the absence of a conceptus [2]. Regardless of the mechanism of initiation, endometrial decidualization is maintained in an embryo-dependent manner. A variety of pregnancy disorders, including infertility, recurrent pregnancy loss (RPL) [3], implantation failure [4], utero-placental disorders, endometriosis [5] and pre-eclampsia [6], have been associated with impaired decidualization.

The steroid hormones estrogen and progesterone, endometrial autocrine and paracrine factors, and miRNAs have been implicated in decidualization, although how these molecules coordinate an endometrial response remains unclear [7, 8]. In addition to nuclear receptors (e.g., progesterone receptor (PR)), some transcription factors have been identified as critical regulators of decidualization, including homeobox transcription factors [9], CCAAT/enhancer binding protein-β (C/EBPβ) [10], and members of the O subclass of the forkhead family of transcription factors (FOXOs) [11]. Sun et al. reported that a zinc finger-containing transcription factor, Krüppel-like factor 5 (KLF5), is expressed dynamically in the luminal epithelium and stroma throughout peri-implantation. KLF5 regulates formation and development of the trophectoderm, inner cell mass, and primitive endoderm, and KLF5^−/−^ mice display embryonic lethality due to developmental defects in the preimplantation embryo. Conditional deletion of KLF5 in the uterus compromises implantation and decidualization [12]. To date, many KLFs, such as KLF12, have been found to participate in steroid hormone signaling and to exhibit pleiotropic actions in uterine endometrial cells [13–15]. KLF12 has been shown to bind to the promoter regions of target genes and represses their expression through an N-terminal PVDLS sequence (Pro-Xaa-Asp-Leu-Ser) that promotes physical interaction with CtBP co-repressors [16]. We have previously demonstrated that KLF12 is expressed in the glandular epithelium (GE) and stromal cells of secretory phase endometrial tissue and that it negatively regulates endometrial decidualization and marker gene transcription [14].

In this study, we showed that KLF12 impaired embryo implantation and endometrial decidualization in mice and that it regulated FOXO1 expression by binding to a CAGTGGG element within the FOXO1 promoter. Moreover, KLF12 was aberrantly expressed in midsecretory endometrial samples from patients with repeated implantation failure (RIF).

## Methods

### Animals and treatments

ICR mice were purchased from the Laboratory Animal Center of Yangzhou University (Yangzhou, China). All animals were bred at the Laboratory Animal Center of Nanjing Drum Tower Hospital (Nanjing, China). All studies were approved by the Institutional Animal Care and Use Committee of Nanjing Drum Tower Hospital (SYXK 2009–0017).

**Fig. 1:**
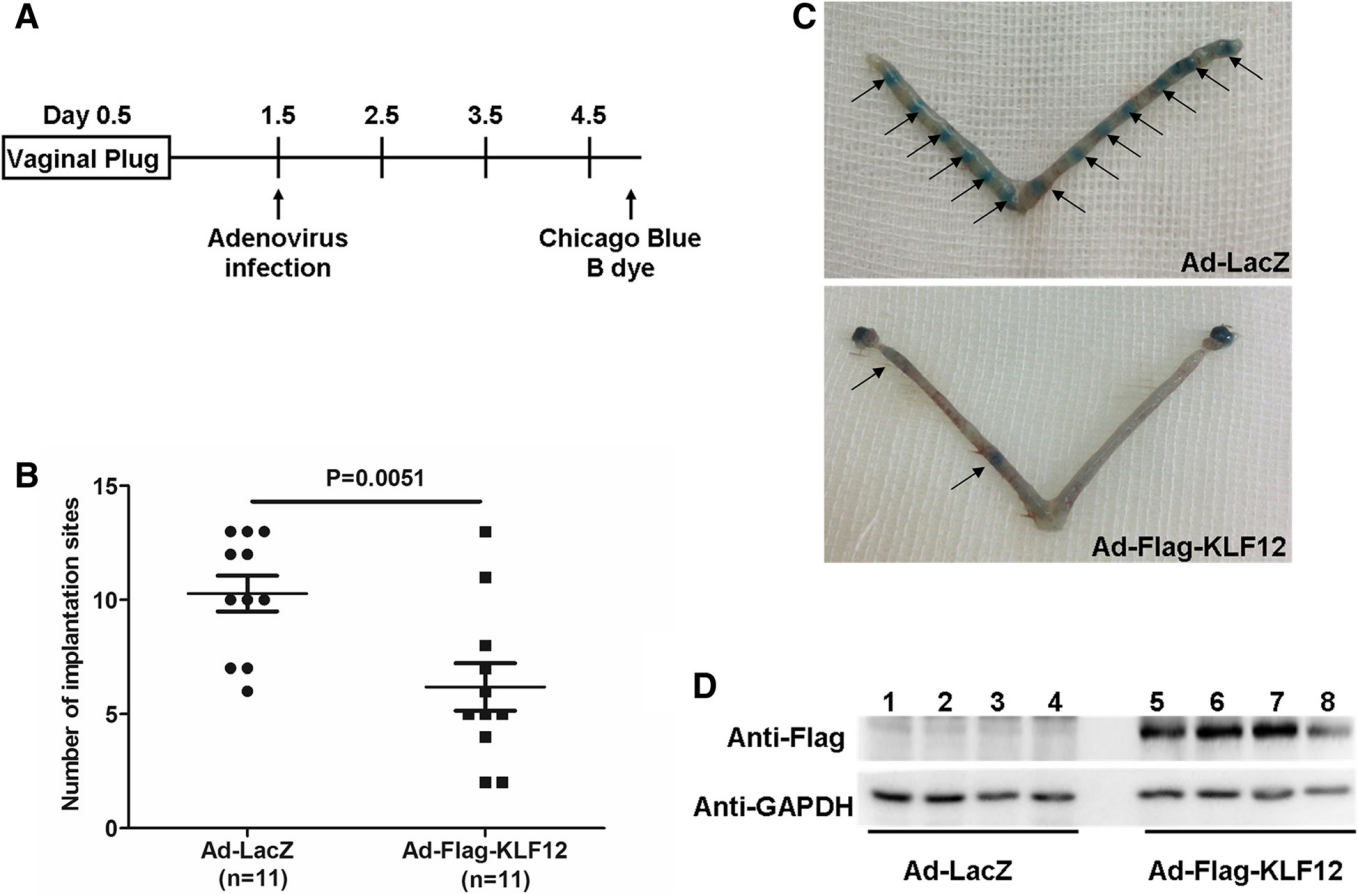
Increased KLF12 expression was associated with impaired implantation *in vivo*. **a** The protocol for detecting the effect of KLF12 on embryo implantation. For specific details, refer to the Methods section (Animals and treatments). **b** The number of implantation sites (ISs) in the afternoon on 4.5 dpc in mice infected with adenovirus. **c** The distinctive banding pattern of a uterine horn stained with Chicago Sky Blue 6B on 4.5 dpc is indicative of an IS in mice treated with Ad-LacZ (*top*) and Ad-Flag-KLF12 (*bottom*). The arrows indicate the locations of the ISs. **d** A representative Western blot of Flag-tag protein in the uteri of the Ad-LacZ-treated (*lanes 1–4*) and Ad-Flag-KLF12-treated (*lanes 5–8*) mice. GAPDH was used as a loading control

Females (six weeks old) were mated with fertile males, and the morning on which a vaginal plug was observed was designated as day 0.5 of pregnancy. Twenty microliters (2 × 10^8^ TU/side) of Ad-LacZ or Ad-Flag-KLF12 adenovirus (packaged, amplified and purified as previously reported [14]) was injected into the bilateral uterine horns of the mice in the morning on 1.5 days post-coitus (dpc). Then, the mated mice were intravenously injected with Chicago Sky Blue 6B (Sigma, St. Louis, MO, USA) in the afternoon on 4.5 dpc to evaluate the state of implantation (Fig. 1a).

**Fig. 2:**
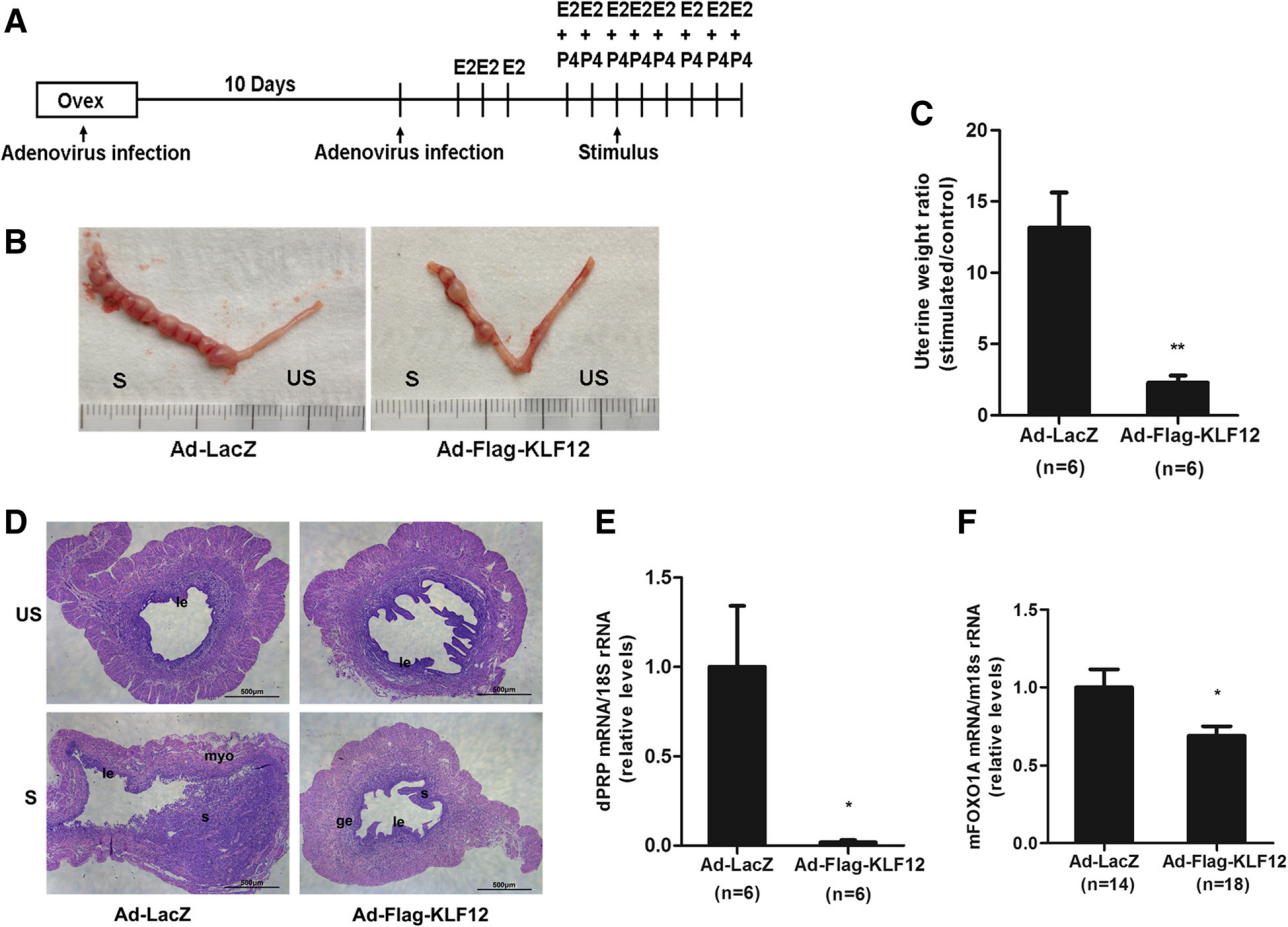
KLF12 overexpression affected decidualization in a mouse model of artificial decidualization. **a** Experimental scheme. Mice were ovariectomized, treated with adenovirus, subjected to an E2/P4 regimen to elicit the estrous cycle, and injected with oil as a decidualization stimulus. The uteri were collected at 5 days after stimulation. **b** Representative uteri after artificial induction of decidualization in the mice. S and US denote the stimulated and unstimulated uterine horns, respectively. **c** The fold changes in the uterine weights of stimulated horns compared with those of unstimulated horns indicates the extent of decidualization in female mice injected with control Ad-LacZ (*n* = 6) or Ad-Flag-KLF12 (*n* = 6) on day 5 after stimulation. **P < 0.01. **d** After artificial induction of decidualization, mouse uterine sections were stained with hematoxylin and eosin. myo, myometrium; s, stroma; le, luminal epithelium; and ge, glandular epithelium. Bar = 500 μm. **e** mRNA expression level of dPRP during *in vivo* decidualization was measured by real-time PCR in the Ad-Flag-KLF12-treated mice (*n* = 6). *P < 0.05 compared with the Ad-LacZ-treated mice (*n* = 6). **f** The FOXO1 mRNA level was measured by real-time PCR in the Ad-Flag-KLF12-treated (*n* = 18) and Ad-LacZ-treated mice (*n* = 14) after 5 days of oil infusion. *P < 0.05

Artificial decidualization was performed as described previously [17]. Briefly, six-week-old mice were subjected to bilateral ovariectomy and treated with 20 μL of Ad-Flag-KLF12 or Ad-LacZ via injection into the bilateral uterine horns (2 × 10^8^ TU/side). Ten days later, the mice received intravenous injection of 20 μL adenovirus (2 × 10^8^ TU/mouse). After two days of rest, the mice were treated with a regimen of E2 and P4 as per the standard protocol and injected with sesame oil into the uterine horns as a decidualization stimulus (Fig. 2a).

### Endometrial sampling and cell culture

All endometrial samples were obtained via endometrial biopsy between days 19 and 23 of the menstrual cycle from 29 patients who exhibited regular menstrual cycles (28–31 days) and who were not taking any oral contraceptives. The demographic details of the patients are summarized in Table 1. RIF was defined as the failure to achieve pregnancy following a minimum of three fresh or frozen cycles, during which at least four good-quality embryos were transferred to the uterus [18]. The subjects in the control group had undergone no less than one successful cycle and delivery. This study was approved by the Institutional Review Board of Nanjing Drum Tower Hospital.

**Table 1:** Demographic details of the participants in the study of endometrial KLF12 expression

Fertile	FER (*n* = 14)	RPL (*n* = 15)	P
Age (years)	29.5 ± 3.8	32.1 ± 4.3	ns
Body mass index (kg/m^2^)	20.5 ± 2.6	22.2 ± 5.9	ns
P4 on hCG day (pg/mL)	9.7 ± 3.5	9.3 ± 3.2	ns
Endometrial thickness (mm)	10.2 ± 1.1	10.0 ± 1.8	ns
No. of transferred embryos	2 ± 0	6.5 ± 2.6	s

The data are presented as the mean ± SD unless otherwise indicated. A *P* < 0.05 was considered significant

*P4* Serum progesterone concentration

Human endometrial stromal cells (hESCs) were isolated and cultured as described previously, and the purity of the cultured stromal cells was determined by cell immunohistochemistry, as shown in Additional file 1: Figure S1. Decidualization was induced according to a previously published protocol [19, 20].

### Western blotting

Proteins were prepared as previously described [19], and the protein content was measured using the Bradford assay (Bio-Rad Laboratories, Hercules, CA, USA). Equal amounts (20 μg) of protein were separated by SDS-PAGE, and immunoblotting was performed with primary antibodies against FOXO1 (Cell Signaling Technology, Danvers, MA, USA, 1:1000), KLF12 (Santa Cruz Biotechnology, CA, USA, 1:2000) or GAPDH (Bioworld Technology, MN, USA, 1:10000), followed by incubation with a horseradish peroxidase (HRP)-conjugated secondary antibody and Flag-HRP (Sigma, 1:5000). The bands were detected using an enhanced chemiluminescence kit (Amersham Biosciences Corp., Piscataway, NJ, USA), and densitometric analysis of each band was performed with Quantity-one (Bio-Rad) software.

### Quantitative reverse transcription real-time PCR (qRT-PCR)

Total RNA was extracted from primary hESCs and from the mouse uterine and human endometrial samples. RNA was reverse-transcribed and subjected to qRT-PCR analysis. The primers for qRT-PCR were as follows: mPRP, sense 5′-GAGAATGGCTGCTCAGATCC-3′, antisense 5′-GTTCAGGTCCATGAGCTGGT-3′; mFOXO1, sense 5′-TCGTACGCCGACCTCATCA-3′, antisense 5′-CTGTCGCCCTTATCCTTGAAGT-3′; hKLF12, sense 5′-CCTTTCCATAGCCAGAGCAG-3′, antisense 5′-TTGCATCCCTCAAAATCACA-3′; hFOXO1, sense 5′-TCATGTCAACCTATGGCAG-3′, antisense 5′-CATGGTGCTTACCGTGTG-3′; m18S rRNA, sense 5′-CGGACATCTAAGGGCATCAC-3′, antisense 5′-ATGGCCGTTCTTAGTTGGTG-3′; and h18S rRNA, sense 5′-CGGCTACCACATCCAAGGAA-3′, antisense 5′-CTGGAATTACCGCGGCT-3′. The qRT-PCR data were analyzed using the 2^-ΔΔCT^ method, with 18S rRNA as an internal control.

### Immunostaining

After the samples were dewaxed, endogenous peroxidase activity was blocked using freshly prepared phosphate-buffered saline (PBS) containing 0.3 % hydrogen peroxide for 15 min. Antigen retrieval was conducted by autoclaving the samples at 121 °C for 15 min in the presence of EDTA (pH 9.0). The sections were washed with PBS and then incubated with antibodies against KLF12 (1:600 dilution, Santa Cruz Biotechnology) and FOXO1 (1:100 dilution, Cell Signaling Technology) overnight at 4 °C in a humidified chamber. Afterward, the sections were rinsed with PBS and incubated with an HRP-conjugated goat anti-rabbit secondary antibody at 37 °C for 30 min. HRP activity was detected using diaminobenzidine (Invitrogen, Carlsbad, CA, USA), and the sections were counterstained with hematoxylin. Nonspecific rabbit serum was used as a negative control, and mouse kidney (KLF12) and human breast carcinoma tissues (FOXO1) were employed as positive controls.

Measurement of KLF12 and FOXO1 protein expression in the endometrium samples was performed using Image-Pro Plus System 6.0 (Media Cybernetics, Inc., Silver Spring, MD, USA) in a blinded fashion, without knowledge of the tissue source. Quantitative analysis was performed according to the software’s instructions. The representative objective protein staining intensity (indicating the relative expression level) was determined according to the mean and integrated optical density (IOD) of the digital image (×400). Signal density data for the tissue areas were obtained from five randomly selected fields of view and subjected to statistical analysis.

### Plasmid construction and reporter gene assays

The wild-type FOXO1 promoter sequence, which spans from −3050 to −2051 bp relative to the transcription start site (Promoter ID: 11424), was amplified by PCR from hESC genomic DNA using the following primers: 5′-GGCCGGTACCTTACAGGATATTTATAATTTT-3′ and 5′-AGAAAAGCTTGCCTTGCCTTCATTTCTATTC-3′. The PCR product was cloned into a pGL3-basic luciferase reporter plasmid (Promega, USA).

Preconfluent (70 %) hESCs in 12-well plates were infected with Ad-Flag-KLF12 for 8 h and then transfected with 0.6 μg FOXO1-Luc plasmid/well using Lipofectamine 2000 (Life Technologies, USA). After 6 h, the culture medium was changed to DMEM-F12 (Invitrogen, Carlsbad, CA) with or without 8-Br-cAMP and medroxyprogesterone acetate (MPA). The cells were incubated for an additional 48 h and then harvested for preparation of cell extracts. Luciferase activity was measured with a Luciferase Assay System (Promega, Madison, USA), in which Renilla luciferase plasmids were cotransfected as controls to standardize the transcription efficiency. These assays were performed using a Centro XS3 LB 960 luminometer (Berthold Technologies, BW, Germany) according to the manufacturer’s protocol.

### Chromatin immunoprecipitation (ChIP)-PCR

Purified DNA fragments were extracted from hESCs and used as templates for PCR and real-time PCR as described previously [14]. A negative control primer set targeting a sequence distal (−7672 to −7473 bp) to the putative binding site was chosen to monitor binding specificity, with the following sequences: 5′-ACCTGCTGACCCAGTGACTC-3′ and 5′-AGGCTGAGGCAGGAGAATG-3′ (−2766 to −2568 bp). The fold enrichment was calculated as the 2^-ΔΔCT^ value and is presented relative to LacZ (after normalization to the input). Annealing was performed at 57 °C, and the PCR products were electrophoresed on 2 % agarose gels and visualized by GoldView staining.

### Avidin-biotin conjugate DNA precipitation (ABCD) assay

Double-stranded oligonucleotides were designed based on the FOXO1 promoter sequence (−2637 to −2601 bp). The 5′-end of the sense strand was biotinylated, and a mutation was introduced (CAGTGGG to CACAAAG) to remove the specific binding site for KLF12. The following primers were designed: human FOXO1 wild-type: 5′-biotin--TCAGGCTGGAGTGCAGTGGGGCGATCTTGGCTCCGC-3′; human FOXO1 wild-type reverse: 5′--GCGGAGCCAAGATCGCCCCACTGCACTCCAGCCTGA-3′; and human FOXO1 mutant: 5′-biotin--TCAGGCTGGAGTGCACAAAGGCGATCTTGGCTCCGC-3′. hESCs were infected with Ad-LacZ and Ad-Flag-KLF12 (25 MOI) for 48 h and then incubated with 8-Br-cAMP and MPA for an additional 48 h. Cell extracts were harvested and lysed in RIPA buffer. Each double-stranded DNA sample (500 pmol) was incubated with 500 μg of cell extract at 4 °C for 2–4 h, and the protein complexes were pulled down using streptavidin agarose beads (Sigma) in binding buffer (10 mM Tris, pH 8.0; 150 mM NaCl; 0.5 % Triton X-100; 0.5 mM DTT; 0.5 mM EDTA; 10 % glycerol; and 20 μg/mL poly [dI–dC]) containing a protease inhibitor cocktail. The proteins were eluted, separated by SDS–PAGE, and then probed with an anti-Flag-HRP antibody.

### Statistical analysis

Unless stated otherwise, the numerical data are presented as the mean ± SD of at least three independent experiments. Student’s t-test was used to determine the statistical significance between two groups. ANOVA with Bonferroni’s correction was employed for multiple comparisons. Pearson correlation analysis was used to assess the relationship between FOXO1 and KLF12. Differences at *P* < 0.05 were considered significant.

## Results

### KLF12 overexpression in the uterus decreased the embryo implantation rate

Mouse embryo implantation occurs at midnight on 3.5 dpc, as determined based on preimplantation ovarian steroid profiles [21]. The uterine lumens of ICR mice were injected with a KLF12-expressing adenovirus (Ad-Flag-KLF12) or Ad-LacZ on 1.5 dpc (Fig. 1a), and the implantation status was then examined on 4.5 dpc to determine whether KLF12 was a critical regulator of embryo implantation. Adenovirus-mediated overexpression of KLF12 markedly increased Flag-KLF12 fusion protein expression (Fig. 1d). The KLF12-overexpressing mice had fewer implantation sites (6.18 ± 1.03, *n* = 11) compared with the Ad-LacZ group (10.27 ± 0.78, *n* = 11) (Fig. 1b and c), suggesting that KLF12 blocked embryonic implantation.

### Increased KLF12 expression in the mouse uterus repressed uterine decidualization

In a previous study, we demonstrated that KLF12 is a novel negative regulator of the decidual reaction and that KLF12 overexpression in hESCs significantly represses their morphological and biochemical transformation [14]. Based on the observations shown in Fig. 1, we hypothesized that impaired decidualization may cause KLF12-induced implantation failure; thus, we investigated decidualization in the hormone-primed uteri of ovariectomized mice treated with a well-established regimen of E2 and P4 (Fig. 2a). As expected, morphological analysis of Ad-LacZ control mice revealed thicker uterine horns, indicating the presence of pronounced deciduoma within 5 days after artificial stimulation (Fig. 2b, *left*). In contrast, the uteri of KLF12-overexpressing mice contained thinner and fewer pseudopregnant sacs under identical conditions (Fig. 2b, *right*). As shown in Fig. 2c, the fold changes in the uterine weights of oil-stimulated horns compared with those of control horns indicated that the extent of decidualization was markedly reduced in the KLF12-overexpressing females. Morphometric analysis of uterine tissue sections further demonstrated that the stimulated Ad-LacZ horns were packed with decidual cells, while the KLF12-overexpressing horns contained a markedly reduced number of decidual cells (Fig. 2d). Consistent with these results, the mRNA expression of dPRP, which is a marker of mouse decidualization [22], was significantly reduced by almost 50-fold in the uteri of the KLF12-overexpressing mice (Fig. 2e). Accordingly, increased uterine KLF12 expression was associated with severe impairment of decidualization. Additionally, FOXO1 mRNA was highly expressed in the control uteri but was down-regulated (reduced by >30 %) in the uteri from the KLF12-overexpressing mice (Fig. 2f).

### Elevated KLF12 level led to a decrease in FOXO1 expression

**Fig. 3:**
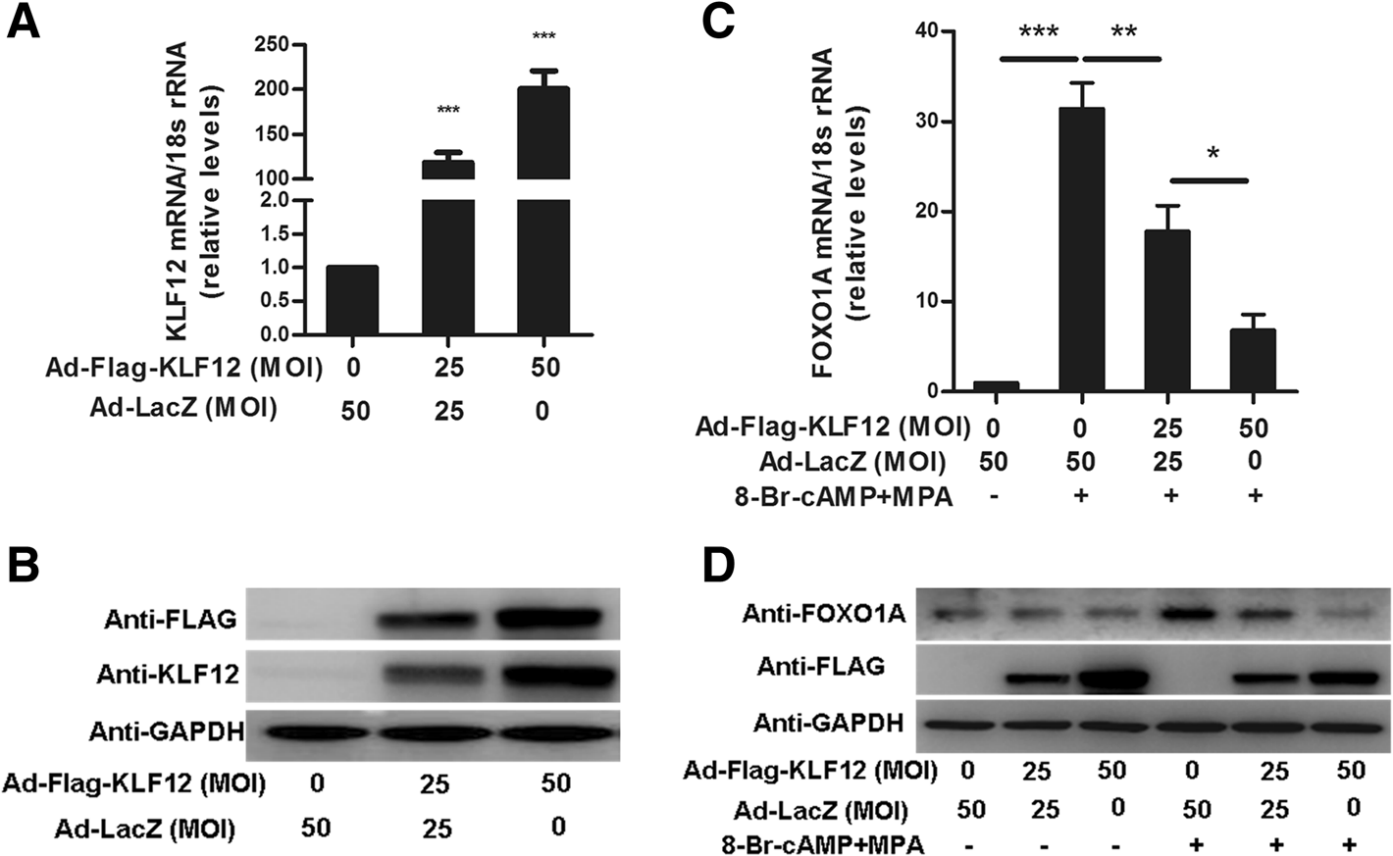
Elevated KLF12 level led to decreased FOXO1 expression. hESCs were infected with adenovirus expressing either LacZ or KLF12 for 48 h at the indicated MOI. The KLF12 transcript copy number (**a**) and its protein expression (**b**) were determined by real-time PCR and Western blotting (*n* = 3). ***P < 0.001 compared with Ad-LacZ alone. hESCs were infected with Ad-LacZ or Ad-Flag-KLF12 at the indicated MOI for 48 h and were then treated with 8-Br-cAMP and MPA for another 2 days; subsequently, FOXO1 mRNA (**c**) and protein (**d**) levels were measured by real-time PCR and Western blotting, respectively (*n* = 3). *P < 0.05; **P < 0.01; and ***P < 0.001

To better understand the mechanism by which FOXO1 expression is regulated, we examined its expression following KLF12 up-regulation in hESCs *in vitro*. As shown in Fig. 3a and b, infection of hESCs with Ad-Flag-KLF12 (MOI = 25 and 50) for 48 h markedly increased KLF12 mRNA expression (by greater than 110-fold and 200-fold, respectively). Importantly, KLF12 overexpression in hESCs resulted in significant dose-dependent decreases in FOXO1 mRNA (Fig. 3c) and protein (Fig. 3d) expression in response to 8-Br-cAMP and MPA.

### KLF12 directly repressed FOXO1 transcription

**Fig. 4:**
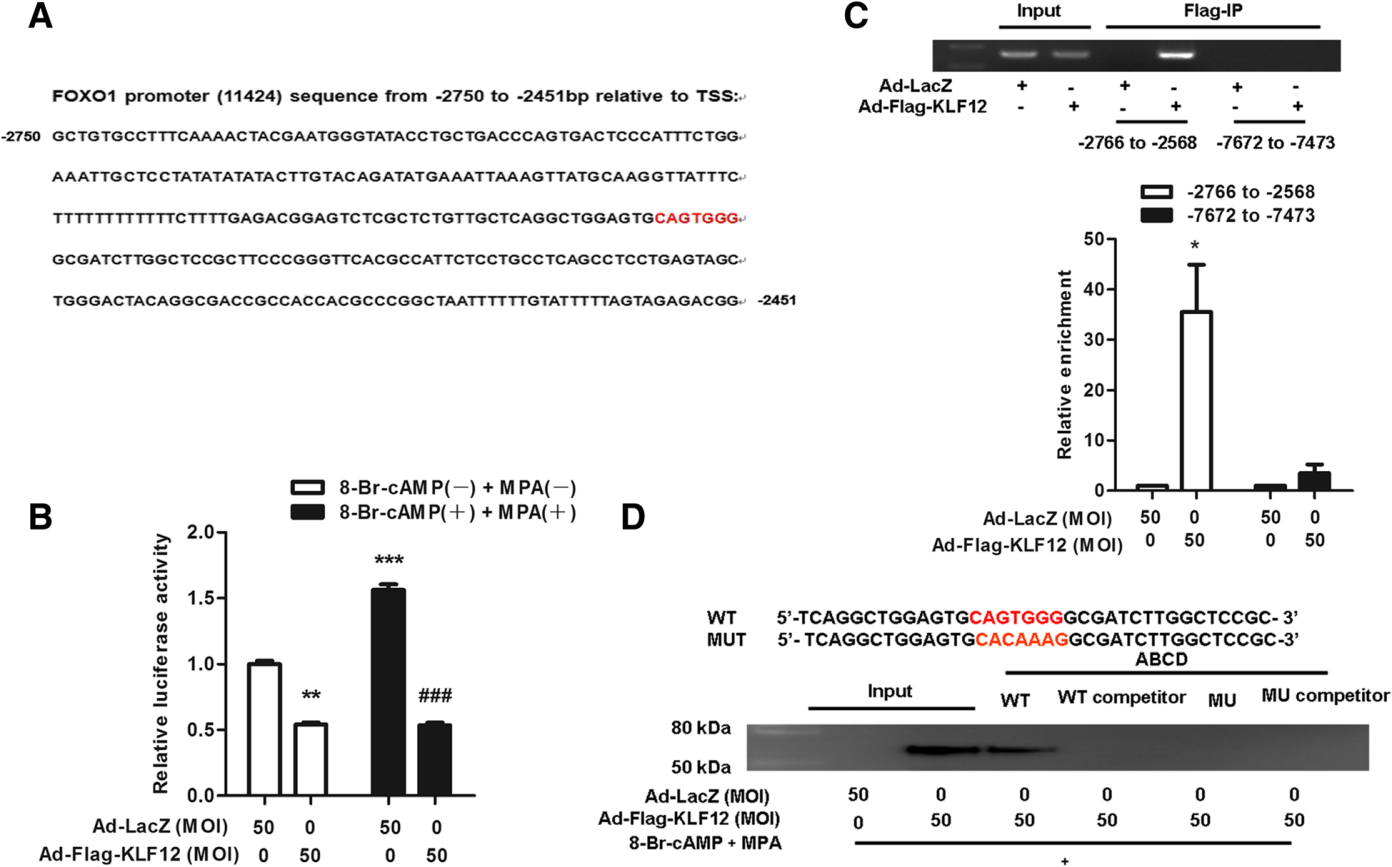
KLF12 mediated the transcriptional repression of FOXO1. **a** Schematic of the location of the KLF12 binding site in the FOXO1 promoter. **b** hESCs were infected with the indicated adenoviruses for 8 h, transfected with FOXO1-LUC (600 ng/well), and treated with 8-Br-cAMP and MPA. After 48 h, luciferase assays were performed, and the data were plotted after normalization to Renilla luciferase activity (*n* = 3). **P < 0.01 and ***P < 0.001 compared with Ad-LacZ without 8-Br-cAMP or MPA; ###*P* < 0.001 compared with Ad-LacZ with 8-Br-cAMP and MPA. **c** ChIP-PCR amplification using primers against the human FOXO1 promoter region (*top*). PCR products were separated by agarose gel electrophoresis. Quantitative ChIP analysis was performed by real-time PCR. The results are shown as the fold enrichment over LacZ (after normalization to the input, *bottom*). Input (non-precipitated) chromatin was utilized as a positive control for these analyses. *P < 0.05. **d** ABCD assays were performed using biotinylated or non-biotinylated (competitor) double-stranded FOXO1 wild-type (WT) and mutant (MUT) oligonucleotides with whole-cell extracts from hESCs

We identified a conserved KLF12 binding element (CAGTGGG) within the FOXO1 core promoter region similar to a human FOXO1 promoter sequence deposited in transcriptional regulatory element database (Promoter ID: 11424, Fig. 4a). Exogenous hormone-induced decidual transformation increased FOXO1A promoter activity, as evidenced by luciferase reporter assays, and this increase was repressed by KLF12 overexpression (Fig. 4b). Next, ChIP-PCR assays showed that KLF12 bound to the FOXO1 promoter region (−2637 to −2601 bp), which contains the conserved CAGTGGG element; however, no PCR product was obtained from the negative control region (−7672 to −7473 bp; Fig. 4c, *top*). Quantitative ChIP revealed that the primers were effectively (greater than 35-fold) amplified from Flag-KLF12 protein immunoprecipitates but not from LacZ control immunoprecipitates (Fig. 4c, *bottom*). Moreover, Flag-tagged KLF12 protein strongly bound to the FOXO1 promoter containing the CAGTGGG sequence but not to the mutated probe (Fig. 4d). These data indicate that FOXO1 is a novel target gene of KLF12.

### KLF12 and FOXO1 expression was aberrant in the endometria of patients with RIF

**Fig. 5:**
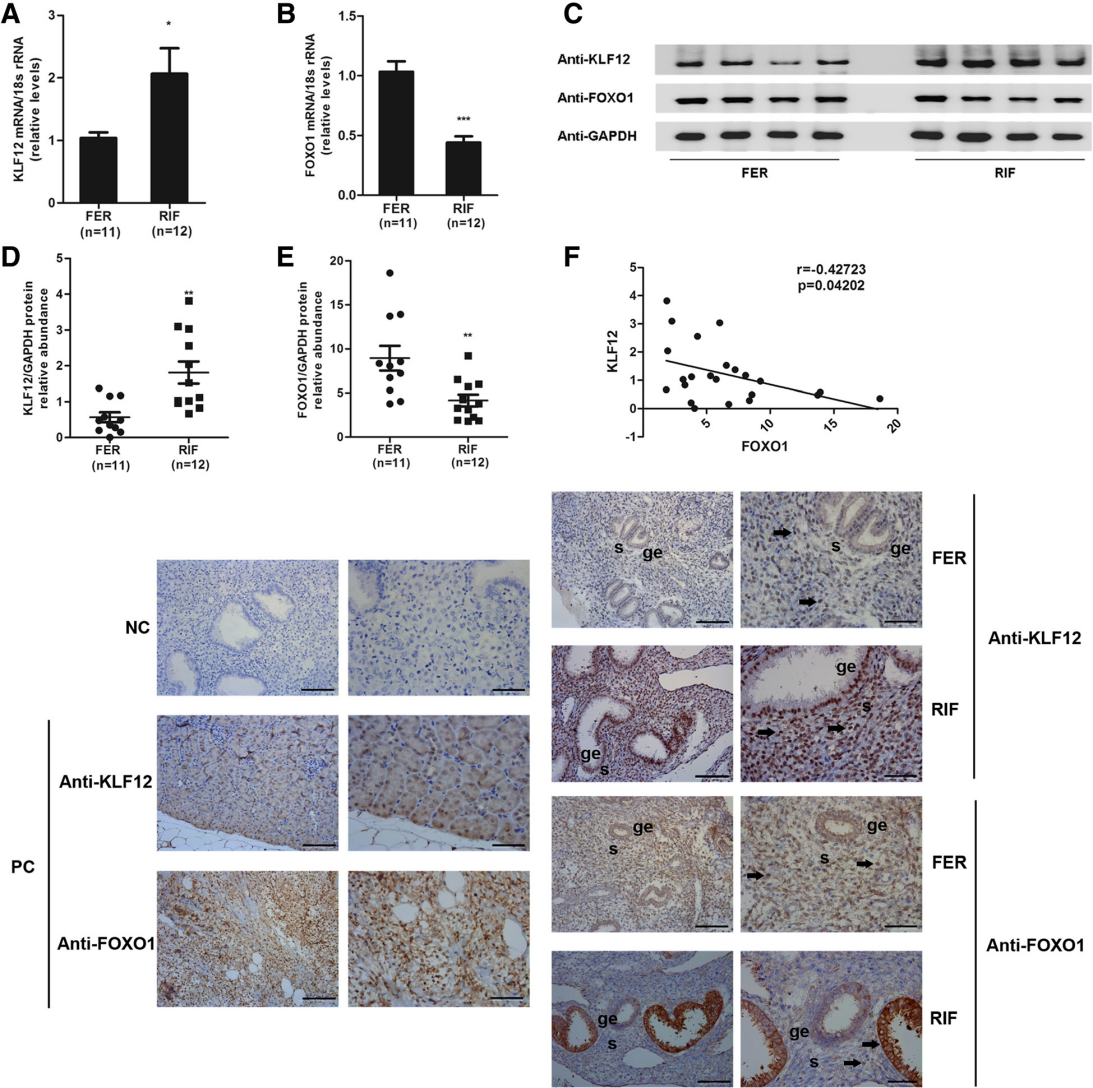
Detection of KLF12 and FOXO1 expression in patients with RIF. The KLF12 (**a**) and FOXO1 (**b**) transcript levels in the uteri of fertile (*n* = 11) and RIF (*n* = 12) patients were quantified by real-time PCR and were then normalized to control 18S gene expression. The data are presented with respect to the fertile group (expression was set to 1). *P < 0.05 and ***P < 0.001 compared with the fertile group. FER, normal fertility women. **c** The difference in protein expression in endometrial samples was assessed by Western blotting using antibodies specific to KLF12 and FOXO1. Total KLF12 (**d**) and FOXO1 (**e**) protein levels were normalized to GAPDH expression, and the data for all of the endometrial samples are shown in the scatter plots. **f** Correlation between KLF12 and FOXO1 protein expression (*n* = 23, *P* < 0.05). **g** Immunohistochemical analysis using KLF12 and FOXO1 antibodies. Endometrial tissue samples from fertile women and those with RIF are shown at 200× (*left panel*) and 400× (*right panel*) magnification. The negative control (NC) is nonspecific rabbit serum, and the positive controls are mouse kidney tissue (KLF12) and human breast carcinoma tissue (FOXO1), respectively. Brown represents positive staining (*arrows*). Scale bar, 100 μm (*left panel*) and 50 μm (*right panel*)

Because the decidual response of hESCs is disrupted in patients with RIF [23], we further assessed the expression of KLF12 and FOXO1 in midsecretory endometrial samples from women with RIF (Table 1). The endometrial KLF12 transcript and protein levels were higher (greater than 2-fold) in women with RIF compared with those in fertile controls (Fig. 5a, c and d). Conversely, expression of FOXO1 was, as expected, significantly reduced by 50 % in the women with RIF compared with that in the fertile controls (Fig. 5b, c and e). The protein level of FOXO1 was moderately negatively correlated with that of KLF12 (*r* = −0.4272395, *P* = 0.04202). Immunohistochemical analysis revealed that the KLF12 and FOXO1 protein levels were higher and lower, respectively, in the endometria of patients with RIF compared with those in the endometria of the fertile controls, particularly in the stromal compartment (Fig. 5g). We quantified the KLF12 and FOXO1 expression densities in the endometrium using Image-Pro Plus System 6.0 image analysis software (Table 2). The IOD of KLF12 for the fertile controls was 232,246 ± 59,354, while that for the patients with RIF was 357,937 ± 86,246; in addition, the mean KLF12 expression density of the patients with RIF (0.38 ± 0.05) was higher than that of the controls (0.31 ± 0.06). The results also showed that the IOD of FOXO1 for the normal controls (609,115 ± 198,330) was significantly higher than that for the patients with RIF (285,440 ± 117,645). The mean densities for the fertile and RIF individuals were 0.34 ± 0.08 and 0.27 ± 0.05, respectively. The results of IHC assay shown in Fig. 5g demonstrate that FOXO1-immunopositive epithelial cells could still be detected in the patients with RIF. We further calculated the level of FOXO1 expression in GE cells. The IOD was 178,465 ± 88,230 (FER, fertile controls) vs. 271,035 ± 119,150 (RIF), and the mean density was 0.23 ± 0.06 (FER) vs. 0.20 ± 0.04 (RIF) (Table 3). In GE cells, the IOD of KLF12 for the RIF group was higher than that for the normal group. These results suggested that increased KLF12 expression in the stromal compartment, together with decreased FOXO1 expression, contributed to the impaired decidualization observed in the women with RIF.

**Table 2:** The means and integrated optical densities of KLF12 and FOXO1 expression in endometrial tissue

Fertile	FER (*n* = 6)	RPL (*n* = 6)	P
Mean density of KLF12 expression	0.31 ± 0.006	0.38 ± 0.05	<0.0001
KLF12 (IOD)	232,246 ± 59,354	357,937 ± 86,246	<0.0001
Mean density of FOXO1 expression	0.34 ± 0.08	0.27 ± 0.05	0.0403
FOXO1 (IOD)	609,115 ± 198,330	285,440 ± 117,645	0.0014

The data are presented as the mean ± SD. A *P* < 0.05 was considered significant, vs. normal fertility women

**Table 3:** The area and integrated optical density of FOXO1 expression in GE of endometrium

Fertile	FER (*n* = 6)	RPL (*n* = 6)	P
Mean density of KLF12 expression	0.12 ± 0.04	0.15 ± 0.05	0.0282
KLF12 (IOD)	71,421 ± 63,423	134,775 ± 87,047	0.0021
Mean density of FOXO1 expression	0.23 ± 0.06	0.20 ± 0.04	0.0012
FOXO1 (IOD)	178,465 ± 88,230	271,035 ± 119,150	0.0290

The data are presented as the mean ± SD. A *P* < 0.05 was considered significant, vs. normal fertility women

## Discussion

This study has verified the important role of KLF12 in the induced decidualization in mice and is the first report of a putative molecular mechanism for the transcriptional repression of FOXO1 in hESCs. KLF12 belongs to the zinc finger-containing transcription factor family, which contains key mediators of endocrine/metabolic processes that play emerging key roles in human reproductive and uterine diseases [15, 24]. For example, KLF11 is highly expressed in human urogenital tissues, and aberrant KLF11 expression has been linked to the pathogeneses of common uterine diseases, such as endometriosis and leiomyoma [25, 26]. Moreover, loss of coregulation between KLF9 and PR may underlie the pathogenesis of endometriosis [27]. Furthermore, KLF9 deficiency in the uterus has been shown to promote the establishment of ectopic lesions in a mouse model of endometriosis [28]. Finally, KLF13-null lesions contribute to defective steroid hormone receptor signaling in the pathology of endometriosis [29], and KLF15 negatively regulates E2-induced epithelial cell proliferation, which is associated with endometriosis and endometrial cancer [30]. All of these reproductive diseases in which KLFs have been implicated are associated with impaired decidualization. Similarly, in this study, increased KLF12 expression led to a severe reduction in endometrial decidualization and blockage of embryo implantation in the endometrium.

KLF12 (AP-2rep) was first identified based on its ability to bind to and down-regulate an important transcription factor, AP2α [31]. Shen et al. found that PRL is a target gene of KLF12, which directly binds to the CAGTGGG sequence in the PRL promoter [14]. In the present study, we similarly identified a novel translational target of KLF12 in endometrial biology. FOXO1 is essential for female reproduction and is markedly induced upon decidualization by both the PKA/cAMP pathway and ligand-activated nuclear PR [32]. FOXO1 physically associates with HOXA10 or PR to modulate the expression of decidua-specific genes, such as insulin-like growth factor-binding protein 1 (IGFBP1) [33, 34]. Furthermore, KLF12 not only transcriptionally represses PRL expression but also reduces the IGFBP1 protein level during decidualization; however, the mechanism by which this regulation occurs remains unclear [14]. The KLF12-mediated repression of FOXO1 expression is potentially the primary cause of impaired decidualization and subsequent implantation failure. Because FOXO1 directly regulates IGFBP1 but KLF12 does not, the mechanism underlying the KLF12-mediated inhibition of IGFBP1 expression via FOXO1 during decidualization must be further addressed.

Although assisted reproductive technologies have markedly reduced the incidence of infertility, 45–60 % of patients undergoing assisted reproduction do not experience a live birth after 3 treatment cycles [35]. Endometrial receptivity is responsible for RIF in approximately two-thirds of affected women [36], and stromal decidualization is considered to be important for endometrial receptivity [4].

As suggested by immunohistochemical analysis of the endometrium of women with RIF, KLF12 expression is elevated in stromal cells as well as in the GE. Interactions between the blastocyst and epithelia and those between epithelial cells and stromal cells are vital to successful implantation [37, 38]. Paracrine signals from the blastocyst result in the production of various hormones and cytokines (e.g., chorionic gonadotropin and interleukin-1) that facilitate attachment and maintenance of pregnancy [39, 40]. After blastocyst attachment, luminal epithelial cells transmit signals to mediate the proliferation and differentiation of the endometrium and to elicit changes in vascular permeability and embryo migration across the epithelium [41, 42]. The GE, another part of the endometrial epithelium, is embedded within the stromal bed. Gland-derived signaling influences stromal function in a paracrine manner [43]. Many products secreted from the GE, such as leukemia inhibitory factor, appear to support nascent embryos and normal stromal decidualization during implantation [44, 45]. Aberrant KLF12 expression in the GE may interfere with crosstalk between the epithelium and stroma, causing hESC decidualization defects. Furthermore, as an anti-inflammatory regulator, KLF12 is down-regulated in patients with aneurysm [46], and decidualization and embryo implantation are usually considered to involve proinflammatory responses. The higher epithelial KLF12 expression observed during implantation in women with RIF indicates that the product of this gene may influence the immuno-inflammatory reaction in the uterine epithelium. Interestingly, in our study, the mean density of FOXO1 in the GE of the control group was slightly higher than that in the GE of the RIF group, while the IOD was weaker in the control group than that in the RIF group. These differences were associated with a significant change in the number of GE cells, as well as slightly altered KLF12 expression in these cells. On the other hand, FOXO1 is required for programmed apoptosis of the epithelium and for safeguarding endometrial homeostasis during the reproductive phase [11]. Additionally, FOXO1 may be regulated by factors other than KLF12, such as miRNAs, in endometrial epithelial cells [47].

KLF12 is involved in defective decidualization in patients with RIF; however, the mechanism by which it is up-regulated remains unclear. Compromised P4 signaling might be responsible for the abnormal endometrial gene expression observed in women with RIF. Thus, the outcomes of RIF may be ascribed to abnormal P4-sensitive maternal signaling rather than to the amount of progesterone, given the similar levels of secreted progesterone in the patients and controls observed in this study. MPA is a synthetic progesterone that interacts with PR to promote differentiation of the uterine endometrium, and in our study, MPA repressed KLF12 expression in hESCs in a time-dependent manner (data not shown). Rubel et al. reported that many members of the KLF family, such as KLFs 3, 4, 7, and 12, are candidate mediators of PR function in P4-responsive target tissues [48]. Therefore, KLF12, a potential P4-regulated gene, may be up-regulated by compromised P4 signaling in the endometria of patients with RIF.

This study constitutes the first functional analysis of the role of KLF12 in endometrial decidualization and embryo implantation *in vivo*. To prevent traumatic adenovirus infection of the uterine horns, a mouse uterine model with a conditional KLF12 knock-in will be employed in future studies to unravel the physiological role and mechanism of action of KLF12 in pregnancy.

## Conclusions

Taken together, our results indicate that KLF12, by negatively regulating FOXO1 expression, contributes to improper stromal decidualization and reduced embryo implantation. These findings may inspire the development of new therapeutic regimens for patients with RIF and disrupted decidualization.

## Additional file

Additional file 1: Figure S1. Cellular immunohistochemical staining of hESCs. (DOCX 617 kb)

## Abbreviations

C/EBPβ: CCAAT/enhancer-binding protein β
E2: Estrogen
FER: Fertility
FOXO1: Forkhead box class O1
FOXOs: O subclass of the forkhead family of transcription factors
GE: Glandular epithelium
hESC: Human endometrial stromal cell
HRP: Horseradish peroxidase
IGFBP1: Insulin-like growth factor-binding protein 1
IOD: Integrated optical densities
IS: Implantation site
IVF: *In vitro* fertilization
KLF5: Krüppel-like factor 5
KLF12: Krüppel-like factor 12
MPA: Medroxyprogesterone acetate
MUT: Mutant
NC: Negative control
P4: Progesterone
PC: Positive control
PR: Progesterone receptor
PRL: Prolactin
PRP: Prolactin-related protein
RIF: Repeated implantation failure
RPL: Recurrent pregnancy loss
S: Stimulated uterine horns
US: Unstimulated uterine horns
WT: Wild type
