# Supplementary material for: Krüppel-like factor 12 is a novel negative regulator of forkhead box O1 expression: a potential role in impaired decidualization
Source: Reprod Biol Endocrinol. 2015 Jul 30;13:80. doi: 10.1186/s12958-015-0079-z (PMC4520059; doi:10.1186/s12958-015-0079-z)
Supplement: Additional file 1: Figure S1. — Cellular immunohistochemical staining of hESCs. (DOCX 617 kb) [file 12958_2015_79_MOESM1_ESM.docx]

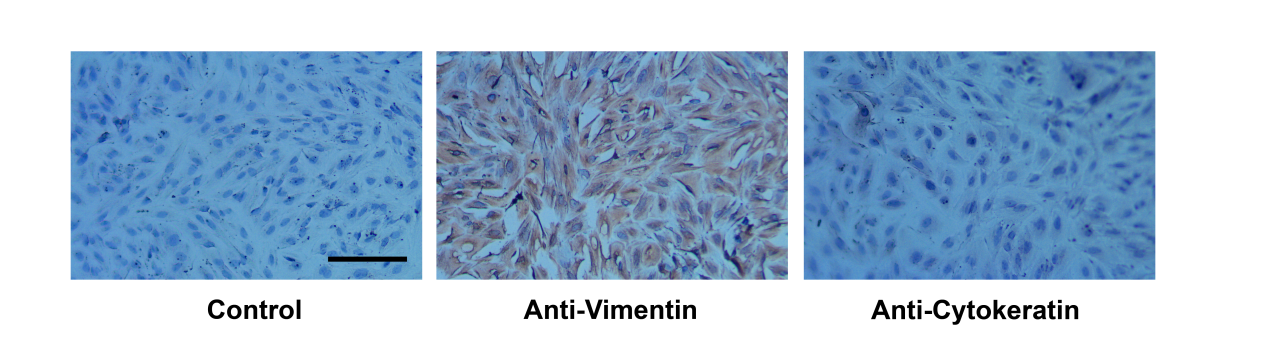


Figure S1: Immunohistochemistry were stained with polyclonal antibodies against vimentin (Epitomics, CA, USA, 1:500) and cytokeratin (Santa Cruz Biotechnology, 1:100). The cells were used between passages 2 in this study. Bar = 200μm.
